# Supplementary material for: The genome sequence of a rove beetle, Tachyporus hypnorum (Fabricius, 1775) (Coleoptera: Staphylinidae)
Source: Wellcome Open Res. 2026 Mar 19;11:182. [Version 1] doi: 10.12688/wellcomeopenres.26176.1 (PMC13080330; doi:10.12688/wellcomeopenres.26176.1)
Supplement: Peer review history

# Reviewer report: Reviewer response for version 1

**DOI:** 10.21956/wellcomeopenres.28829.r151926

**Published:** 2026-04-14

**Authors:** Nikoletta Andrea Nagy

**Affiliations:** 1 University of Debrecen, Debrecen, Hungary

The manuscript is well written, and the genome presented can contribute to future research on rove beetles. I have only a few suggestions for the authors.

 Background

 - when describing the appearance species, I would recommend changing "It is" to "The body of the species"

 - suggested change:

     from "Its distinctively coloured pronotum, general shape and size mean that, with experience, it can be recognised in the field"

     to "Based on its distinctively coloured pronotum, general shape and size, the species can be recognised in the field"

 Genome assembly

 - please include the read filtering method

 Sequence data

 - the haploid genome size in the main text does not match with the one on Figure 2

 Assembly statistics

 - please include whether the assembled mitochondrial genome was circularized or linear

Are sufficient details of methods and materials provided to allow replication by others?

Yes

Is the rationale for creating the dataset(s) clearly described?

Yes

Are the datasets clearly presented in a useable and accessible format?

Yes

Are the protocols appropriate and is the work technically sound?

Yes

Reviewer Expertise:

genome assembly, genome annotation, transcriptome analysis

I confirm that I have read this submission and believe that I have an appropriate level of expertise to confirm that it is of an acceptable scientific standard.

# Reviewer report: Reviewer response for version 1

**DOI:** 10.21956/wellcomeopenres.28829.r151927

**Published:** 2026-04-14

**Authors:** Wai Lok So

**Affiliations:** 1 The Chinese University of Hong Kong, Hong Kong, Hong Kong

The manuscript presents a genome assembly of T *achyporus hypnorum* as a part of the Darwin Tree of Life initiative. The assembly methods are technically sound, yielding a genome with 13 pseudo-chromosomes, including the sex chromosome and a complete mitochondrial genome. Below are some comments for the improvement of the current manuscript.

 Comments:

 1. The authors failed to mention the importance of sequencing this beetle species. It is encouraged the authors to state the rationale for creating such genome resources in the Background.

 2. The authors spent a paragraph in Background in illustrating the morphology of the beetle, which is detailed and sufficient. However, the orientation and appearance of the beetle photograph is poor in Figure 1. It gives no clue on the identity of the described beetle. Could the authors also provide a proper and nicer photo for this species in Figure 1?

 3. The authors mentioned the sample was dissected and stored in ethanol in Methods. Could the authors provide more information/details on the dissection? What had been dissected and what kind of tissues had been taken out? How much was the tissue? Was the gut remove in the sample?

 4. The authors mentioned that they used the centrifuge-mediated fragmentation to produce the DNA fragments in 8-10 kb range. Please kindly provide the profile of the centrifuge in achieving the desired fragment sizes.

 5. The authors generated a genome with 13 chromosomes from 63.74% of assembled sequences. Does this chromosome number match the other beetle genomes in the same family or genus level? There are still a lot of unmapped sequences. Could the authors identify those sequences? Are there any genes found on them?

 6. There are some formatting issues that need to be polished, e.g. spacing and numerical value presentation. Please kindly proofread.

Are sufficient details of methods and materials provided to allow replication by others?

Partly

Is the rationale for creating the dataset(s) clearly described?

No

Are the datasets clearly presented in a useable and accessible format?

Yes

Are the protocols appropriate and is the work technically sound?

Yes

Reviewer Expertise:

evolution, genomics, arthropods, myriapods

I confirm that I have read this submission and believe that I have an appropriate level of expertise to confirm that it is of an acceptable scientific standard, however I have significant reservations, as outlined above.

# Reviewer report: Reviewer response for version 1

**DOI:** 10.21956/wellcomeopenres.28829.r151925

**Published:** 2026-04-08

**Authors:** Henrik Lantz

**Affiliations:** 1 SciLifeLab, Uppsala University, Uppsala, Sweden

This manuscript presents the genome sequence of a small beetle, Tachyporus hypnorum, assembled as a part of the Darwin Tree of Life project. The species is widespread in Europe and the specimens used here were sampled in England. Despite being only 3-4 mm in length, the species is distinctive enough to be, with experience, identified in the field.

 The genome is the first high-quality genome assembled for the genus and thus constitutes an important resource not only for studies of the species but also for comparative studies at higher taxonomical levels. Methods are well-described and follow a high international standard. The assembly is of high quality and is certainly useful for most studies, including looking at genomic rearrangements which requires high contiguity, but fails to meet Earth Biogenome Project standards as only 63.74% of the assembly could be assigned to chromosomes (should be at least 90%).

 I have two main comments and a small one, all intended to improve clarity and transparency.

 1. It is not clearly stated what was done to facilitate re-confirmation of the identity of the specimens used in the study. Because of the small size of the species, whole individuals needed to be used and this of course limits what can be preserved, but this should then be explained in the text. If someone wants to confirm the identity of the specimens used, for example if a need arises to describe sub-species, what remains for that researcher to base conclusions on? Was anything at all saved from the specimens, and where is that material stored in that case? Is the photo detailed enough for an expert to base identification on? Where is that photo stored in that case? The use of DNA barcoding to verify the identity of the specimens does strengthen the identification, but is of limited use for within species studies. Remaining voucher material and/or photos should be stored in a Natural History Museum, and enough information should be given in the manuscript to allow for a reader to find that material.

 2. 13 large scaffolds were considered chromosome-level, and this number correlates well with the expected number of chromosomes given in Genomes On A Tree, based on close relatives. However, only 63.74% of the assembly is assigned to these chromosomes, and no effort is done in the manuscript to explain what the remaining 36.26% are. As I understand it, heterochromatin is also known from Coleoptera and can constitute a large part of the genome, and perhaps the unassigned part of the assembly is simply that, but then I would like to see this stated in the manuscript.

 3. Figure 6, BlobToolKit plot: It seems most likely to me that the plot is done after decontamination, but still, it would be nice to have that clearly stated. I also find it likely that the smaller blobs with no hits are the contigs that could not be assigned to chromosomes, but would appreciate if that also could be detailed in the figure text.

Are sufficient details of methods and materials provided to allow replication by others?

Yes

Is the rationale for creating the dataset(s) clearly described?

Yes

Are the datasets clearly presented in a useable and accessible format?

Yes

Are the protocols appropriate and is the work technically sound?

Yes

Reviewer Expertise:

Genome assembly, genome annotation, taxonomy, systematics, phylogeny.

I confirm that I have read this submission and believe that I have an appropriate level of expertise to confirm that it is of an acceptable scientific standard, however I have significant reservations, as outlined above.
